# White Matter Atrophy in Type 2 Diabetes Mellitus Patients With Mild Cognitive Impairment

**DOI:** 10.3389/fnins.2020.602501

**Published:** 2021-01-18

**Authors:** Chang Li, Rongbing Jin, Kaijun Liu, Yang Li, Zhiwei Zuo, Haipeng Tong, Jingna Zhang, Junfeng Zhang, Yu Guo, Yuqi Lai, Jinju Sun, Jian Wang, Kunlin Xiong, Xiao Chen

**Affiliations:** ^1^Department of Radiology, Daping Hospital, Army Medical University, Chongqing, China; ^2^Department of Radiology, Southwest Hospital, Army Medical University, Chongqing, China; ^3^Department of Nuclear Medicine, Daping Hospital, Army Medical University, Chongqing, China; ^4^Chongqing Clinical Research Center for Imaging and Nuclear Medicine, Chongqing, China; ^5^Department of Gastroenterology, Daping Hospital, Army Medical University, Chongqing, China; ^6^Department of Radiology, General Hospital of Western Theater Command, Chengdu, China; ^7^Department of Medical Imaging, College of Biomedical Engineering, Army Medical University, Chongqing, China; ^8^School of Foreign Languages and Cultures, Chongqing University, Chongqing, China

**Keywords:** type 2 diabetes mellitus, mild cognitive impairment, white matter volume, magnetic resonance imaging, biomarker

## Abstract

Type 2 diabetes mellitus (T2DM) patients are highly susceptible to developing dementia, especially for those with mild cognitive impairment (MCI), but its underlying cause is still unclear. In this study, we performed a battery of neuropsychological tests and high-resolution sagittal T1-weighted structural imaging to explore how T2DM affects white matter volume (WMV) and cognition in 30 T2DM-MCI patients, 30 T2DM with normal cognition (T2DM-NC) patients, and 30 age-, sex-, and education-matched healthy control (HC) individuals. The WMV of the whole brain was obtained with automated segmentation methods. Correlations between the WMV of each brain region and neuropsychological tests were analyzed in the T2DM patients. The T2DM-NC patients and HC individuals did not reveal any significant differences in WMV. Compared with the T2DM-NC group, the T2DM-MCI group showed statistically significant reduction in the WMV of seven brain regions, mainly located in the frontotemporal lobe and limbic system, five of which significantly correlated with Montreal Cognitive Assessment (MoCA) scores. Subsequently, we evaluated the discriminative ability of these five regions for MCI in T2DM patients. The WMV of four regions, including left posterior cingulate, precuneus, insula, and right rostral middle frontal gyrus had high diagnostic value for MCI detection in T2DM patients (AUC > 0.7). Among these four regions, left precuneus WMV presented the best diagnostic value (AUC: 0.736; sensitivity: 70.00%; specificity: 73.33%; Youden index: 0.4333), but with no significant difference relative to the minimum AUC. In conclusion, T2DM could give rise to the white matter atrophy of several brain regions. Each WMV of left posterior cingulate, precuneus, insula, and right rostral middle frontal gyrus could be an independent imaging biomarker to detect cognitive impairment at the early stage in T2DM patients and play an important role in its pathophysiological mechanism.

## Introduction

Type 2 diabetes mellitus (T2DM) is a common metabolic disorder characterized by insulin resistance and hyperglycemia, which has become a significant health problem throughout the world. It can cause severe multi-systemic dysfunction, such as kidney, eye, peripheral, central nervous system, etc. Epidemiological investigations have shown that T2DM is associated with a twofold increased risk of dementia and can affect a wide range of cognitive abilities (Peila et al., 2002; Biessels et al., 2006; Crane et al., 2013). Mild cognitive impairment (MCI) is considered as a precursor of dementia, which has been an increasingly common target of potential therapeutic trials (Schneider et al., 2009; Brooks and Loewenstein, 2010; Sperling et al., 2011). Currently, the occurrence of dementia is an irreversible process and has no effective cure solution. The effective and preventive treatments are needed at early phases of the dementia spectrum (Jedynak et al., 2012). Thus, the early detection of alterations in T2DM patients with MCI is important for patient care and developing future treatment.

Nowadays, the cognitive tests have been widely used for detection of cognitive impairment. However, the cognitive tests scores could be affected by the subjective judgment and drug intervention. Brain magnetic resonance (MR) imaging provided a good opportunity to explore the diabetic cerebral changes. The MR imaging biomarkers are more objective evidence, which could provide a supplement and clue for early diagnosis of cognitive impairment in T2DM patients. On conventional MR images, brain atrophy in some regions could be found (de Bresser et al., 2010; van Elderen et al., 2010; Espeland et al., 2013; Moran et al., 2019). Recently, lots of attention have been paid on the gray matter changes of T2DM patients. Several studies have shown that regional atrophy patterns of gray matter mainly located in frontal lobe, middle temporal gyrus, and posterior cingulate gyrus (Brundel et al., 2010; Moran et al., 2013; Moulton et al., 2015; Fang et al., 2018; Li et al., 2018). Despite the importance of gray matter atrophy in T2DM, white matter abnormalities played a distinct and irreplaceable role in cognitive impairments induced by T2DM. White matter has a vital role for transferring information between gray matter regions. White matter alterations include both microstructural deficits and morphological abnormalities. On one hand, studies of white matter integrity using MR diffusion tensor imaging (DTI) have demonstrated microstructural alterations in different regions in T2DM patients. These studies observed reduced white matter integrity predominantly in the cingulum, the uncinate fasciculus, the superior and inferior longitudinal fasciculus, corpus callosum, and external and internal capsule (Zhang et al., 2014; Nouwen et al., 2017; Yoon et al., 2017; Sun et al., 2018; Xiong et al., 2019). On the other hand, volumetric measurements are widely used to study morphological changes in both normal aging and in neurodegenerative disorders. Understanding changes in regional brain volumes has the potential to aid prediction of onset and progression of many neurodegenerative disorders. However, few studies have been focused on alterations of white matter volume (WMV) in T2DM patients. The loss of WMV in T2DM patients has been observed in temporal lobe, inferior frontal triangle region, and hippocampus (Korf et al., 2006; Gold et al., 2007; Chen et al., 2012; Moran et al., 2013). The alterations of WMV in previous studies did not show a consistent pattern in T2DM. The reason for the inconsistent results may be that T2DM patients included in these studies were in different stages of diabetes-associated cognitive impairment. To our knowledge, we have not found the report of WMV changes in T2DM patients with MCI at present.

In the current study, we enrolled T2DM patients with mild cognitive impairments (T2DM-MCI), T2DM patients with normal cognition (T2DM-NC), and healthy controls (HC) who performed clinical assessment, a battery of neuropsychological tests, and high-resolution sagittal T1-weighted structural MR imaging to explore how T2DM affects white matter and cognition. Automated segmentation analyses were applied to investigate the WMV difference of the whole brain in these three groups. Then, we assessed the relationship between the WMV of each brain region and neuropsychological tests in the T2DM groups. Finally, we identified the imaging biomarkers to detect MCI in T2DM patients. We expect that our study could provide imaging biomarkers and new insight into the neuropathological mechanisms of T2DM-related cognitive impairment.

## Materials and Methods

### Participants

Samples were collected from 90 patients, consisting of 30 HC, 30 patients with T2DM-NC (T2DM-NC group), 30 patients with T2DM with MCI (T2DM-MCI group). Their overall data were accumulated and categorized by gender, age, and years of education. From October 2015 to June 2020, our hospital recruited all the T2DM patients with and without MCI. The World Health Organization’s criteria were adopted to diagnose T2DM. The accurate diagnosis of MCI was made on the basis of the criteria set up in European Alzheimer’s Disease Consortium in 2006, which involves Mini-Mental State Examination (MMSE) score > 24, clinical dementia rating (CDR) ≥ 0.5, MoCA score < 26, normal activities of daily living (ADL) score, and complaints of hypomnesia. Each patient was tested by structural MRI, neurological, and neuropsychological examinations. All the participants signed informed consent before the study started and were right-handed. If participants had a history of brain injury, epilepsy, alcoholism, Parkinson’s disease, major depression, or other psychiatric or neurological disorder, they were excluded. The participants with severe claustrophobia or contraindications to MRI, severe depression (Hamilton Depression Rating Scale ≥ 18), and dementia (MMSE ≤ 24) were also excluded. If patients with T2DM had microvascular complications including nephropathy, retinopathy, and neuropathy, they were excluded. Thirty volunteers with no cognitive complaints or psychiatric illnesses, nervous system diseases, and vascular risk factors were enlisted as the HC. Participant was excluded if white matter hyperintense lesions on fluid-attenuated inversion recovery (FLAIR) imaging were found. The white matter lesions were also evaluated according to a 10-level scale from barely detectable white matter changes (grade 1) to extensive, confluent changes (grade 9) (Manolio et al., 1994; Chen et al., 2012), and grade 0 (normal white matter) and grade 1 (barely detectable white matter changes) were included and other grades were excluded in this study. Each individual was measured for height, weight, and body mass index (BMI). The Medical Ethics Committee of our institution approved this study, which was conducted in accordance with the principles of the Declaration of Helsinki.

### Neuropsychological Assessments

The Montreal Cognitive Assessment (MoCA), MMSE, Rey-Osterrieth Complex Figure Test (ROCF), Trail-Making Test (TMT), Auditory Verbal Learning Test (AVLT), Verbal Fluency Test (VFT), Digit Span Test (DST), Digit Symbol Coding Test (DSCT), and Hamilton Depression Scale (HAMD) were included in the neuropsychological assessments.

### Standard Laboratory Tests

Standard laboratory tests were executed to evaluate glycosylated hemoglobin (HbA1c), fasting plasma glucose (FPG), fasting insulin, fasting C-peptide, high-density lipoprotein (HDL), low-density lipoprotein (LDL), total cholesterol (TC), triglycerides (TG), urinary microalbumin, homocysteine, blood urea nitrogen (BUN), uric acid, cystatin C, serum creatine, free thyroxine (FT), free triiodothyronine (FT3), and thyroid stimulating hormone (TSH).

### MR Image Acquisition

A 3 T Trio MRI system (Siemens Healthcare, Erlangen, Germany) equipped with a 12-channel phase-array head coil was adopted to collect all MRI data. During the image acquisition, the tested subjects were asked not to move and to keep calm with their eyes closed. By using a T1-weighted magnetization prepared rapid acquisition gradient echo (MPRAGE) sequence (repetition time = 1,900 ms, echo time = 2.52 ms, inversion time = 900 ms, flip angle = 9°, matrix = 256 × 256, thickness = 1.0 mm, 176 slices with voxel size = 1 × 1 × 1 mm^3^), the 3D high-resolution structural images were procured. Afterward, conventional brain T1-weighted imaging (TR/TE = 200/2.78 ms, flip angle = 70°, matrix = 384 × 384, thickness = 4.0 mm, 25 slices, voxel size = 0.7 × 0.6 × 5 mm^3^) and FLAIR imaging (TR/TE/TI = 9,000/93/2,500 ms, flip angle = 130°, matrix = 256 × 256, thickness = 4.0 mm, 25 slices, voxel size = 0.9 × 0.9 × 4 mm^3^) were subjected to all the subjects to exclude white matter hyperintense lesions and organic diseases.

### Image Processing

The data were outputted to a personal computer from the MRI scanner. Off-line analysis was carried out and Linux Operating System was the operating system. Firstly, all the images were confirmed not affected by head motion prior to further analysis of the 3D brain images. Afterward, we converted all the data to MGZ (compressed Massachusetts General Hospital file) format. Meanwhile, by using FreeSurfer software (version 5.3.0, available at http://surfer.nmr.mgh.harvard.edu), the whole brain WMVs were measured. There were multiple steps of the automated processing stream of FreeSurfer, which contained Talairach transformation, removal of non-brain tissue, automatic correction of topological defects, inflation of the folded surface, and registration into an average spherical surface template. In order to segment the gray/white matter tissue and CSF with sub-millimeter precision, a deformable surface algorithm was utilized. Raw volumes for the total WMV were extracted as well as segmented volumes of 68 WMVs (34 regions in each hemisphere) in white matter regions (based on Killiany-Desikan atlas) (Harrisberger et al., 2018; Guo et al., 2019).

### Statistical Analyses

Statistical analyses were performed using SPSS software (version 20.0; IBM Corp., Armonk, NY). The data distribution was verified using the Kolmogorov–Smirnov test. For WMV, comparisons among the three groups were performed by using ANOVA test, with the level of significance setting at *P* < 0.05, false discovery rate (FDR) corrected. For demographics and neuropsychological testing, comparisons among the three groups were performed by using the analysis of variance (ANOVA) test, if the data distribution was normal. The level of significance for intergroup differences was set at *P* < 0.05. The chi-squared analyses were applied to nonparametric analyses if the data distribution was not normal. *Post hoc* tests with Bonferroni correction were performed after observing statistical differences among the three groups. *P* < 0.017 (0.05/3) was considered significant after Bonferroni correction. For the T2DM patients, the correlations between the WMV of each brain region and the neuropsychological scale scores were tested using Pearson correlation analyses. For receiver operating characteristic (ROC) analysis, areas under the curves (AUCs) were used to evaluate the diagnostic value of each marker. Generally, an AUC greater than 0.9 indicated excellent diagnostic efficacy, between 0.7 and 0.9 indicated good diagnostic efficacy, between 0.5 and 0.7 indicated poor diagnostic efficacy, and no more than 0.5 indicated the lack of a diagnostic value. MedCalc Statistical Software (version 19.3.1; MedCalc Software Ltd, Ostend, Belgium) was used to compare differences in AUCs. *P* < 0.05 was considered statistically significant.

## Results

### Demographics and Neuropsychological Testing

The demographic, clinical, and neuropsychological data for the T2DM-MCI, T2DM-NC, and HC groups are shown in Tables 1, 2. No significant intergroup difference was observed in age, gender, years of education, systolic, or diastolic blood pressure, LDL, total cholesterol, and triglycerides. T2DM-NC patients presented higher FPG and HbA1c and lower HDL than HC (Bonferroni correction, *P* < 0.017); T2DM-MCI patients presented higher BMI, FPG, and HbA1c and lower HDL than HC (Bonferroni-correction, *P* < 0.017), while no significant differences were found between T2DM-NC and T2DM-MCI patients. As expected, cognitive function evaluated by various neuropsychological tests showed the worst performance in T2DM-MCI patients than HC and T2DM-NC patients. No significant difference was shown between T2DM-NC patients and HC (Bonferroni correction, *P* > 0.017). Furthermore, T2DM-MCI patients exhibited significant decreases in multiple domains of cognitive function including episodic memory, working memory, executive function, and attention domains when compared with T2DM-NC patients and HC, but no differences in language ability and spatial processing.

**TABLE 1:** Demographic and clinical data of the subjects.

	T2DM-MCI	T2DM-NC	HC	*F*-value (*t*/χ^2^)	*P-*values
Numbers	30	30	30	–	–
Age (years)	55.9 ± 6.54	54.97 ± 5.54	53.17 ± 6.57	1.491	0.231
Sex (male/female)	12/18	19/11	14/16	3.467	0.177^a^
Education (years)	10.43 ± 2.94	11.90 ± 2.92	11.80 ± 2.99	2.316	0.105
Diabetes duration (years)	6.93 ± 5.46	7.93 ± 5.98	–	0.699	0.933
BMI (kg/m^2^)	25.59 ± 3.31	24.08 ± 3.02	23.45 ± 2.59	4.052	0.021*
Systolic blood pressure (mmHg)	130.57 ± 18.25	131.37 ± 14.78	128.13 ± 18.10	0.194	0.749
Diastolic blood pressure (mmHg)	80.60 ± 10.33	82.90 ± 9.80	79.77 ± 9.17	0.895	0.441
Fasting glucose (mmol/L)	9.14 ± 3.05	8.56 ± 1.97	5.47 ± 0.63	25.769	< 0.001^*#^
HbA1c (%)	9.13 ± 2.07	8.84 ± 1.72	5.50 ± 0.36	49.505	< 0.001^*#^
Total cholesterin (mmol/L)	5.24 ± 1.45	4.96 ± 1.37	5.21 ± 0.96	0.432	0.651
HDL cholesterin (mmol/L)	1.16 ± 0.33	1.18 ± 0.30	1.40 ± 0.33	5.167	0.008^*#^
LDL cholesterin (mmol/L)	3.36 ± 1.06	2.93 ± 0.78	3.10 ± 0.67	1.874	0.160

All subjects (T2DM-MCI, T2DM-NC, HC) were matched for age, sex, and education. Values are the mean ± standard deviation. ^*a*^Chi-square test for sex. The comparisons of demographic and clinical data among three groups were performed with ANOVA. The level of significance for intergroup differences was set at P < 0.05. * Denotes P < 0.017 (0.05/3) T2DM-MCI vs. HC with post hoc test, Bonferroni corrected. ^#^Denotes P < 0.017 (0.05/3) T2DM-NC vs. HC with post hoc test, Bonferroni corrected. No significant difference was shown between T2DM-NC and T2DM-MCI with post hoc test, Bonferroni corrected. BMI, body mass index; HbA1c, glycosylated hemoglobin; HDL, high-density lipoprotein; LDL, low-density lipoprotein.

**TABLE 2:** Comparison of the neuropsychological test results among T2DM-MCI, T2DM-NC, and HC groups.

	T2DM-MCI (*n* = 30)	T2DM-NC (*n* = 30)	HC (*n* = 30)	*F*-value (*t*/χ^2^)	*P-*values
**General mental status**					
MoCA	22.93 ± 1.95	27.00 ± 0.83	27.77 ± 1.28	99.370	< 0.001^*#^
MMSE	27.93 ± 1.31	28.43 ± 1.07	28.43 ± 1.17	1.774	0.176
**Episodic memory**					
AVLT-immediately recall	19.50 ± 4.55	22.77 ± 3.83	22.83 ± 5.00	5.406	0.006^*#^
AVLT-delayed recall (5 min)	7.23 ± 2.40	7.80 ± 1.56	8.20 ± 1.94	1.775	0.176
AVLT-delayed recall (20 min)	6.83 ± 2.64	7.33 ± 1.77	7.87 ± 2.18	1.620	0.204
AVLT-recognition	21.10 ± 3.60	21.87 ± 1.48	22.90 ± 1.45	4.252	0.017*
ROCF-immediate recall	19.00 ± 6.64	22.63 ± 6.95	24.08 ± 8.49	3.754	0.027*
ROCF-delayed recall (20 min)	18.20 ± 6.19	21.85 ± 7.13	23.65 ± 7.74	4.659	0.012*
**Working memory**					
WAIS	35.63 ± 8.33	41.23 ± 10.83	46.23 ± 11.99	7.658	0.001*
DST-forward	8.93 ± 1.08	8.87 ± 0.82	9.57 ± 1.38	3.584	0.032
DST-backward	4.67 ± 0.88	5.03 ± 0.89	5.53 ± 1.17	5.805	0.004*
**Spatial processing**					
ROCF-copy	31.97 ± 3.88	32.55 ± 4.11	33.17 ± 1.90	0.911	0.406
**Executive function**					
TMT-B	80.77 ± 26.59	67.73 ± 24.19	61.33 ± 22.74	4.877	0.010*
**Language ability**					
VFT	40.60 ± 7.04	44.10 ± 8.29	44.83 ± 6.06	2.970	0.057
**Attention**					
TMT-A	63.80 ± 21.66	51.97 ± 17.44	48.57 ± 16.61	5.484	0.006^*#^

All subjects (T2DM-MCI, T2DM-NC, HC) were matched for age, sex, and education. Values are the mean ± standard deviation. The comparisons of each neuropsychological test among three groups were performed with ANOVA. The level of significance for intergroup differences was set at P < 0.05. * Denotes P < 0.017 (0.05/3) T2DM-MCI vs. HC with post hoc test, Bonferroni corrected. ^#^Denotes P < 0.017 (0.05/3) T2DM-MCI vs. T2DM-NC with post hoc test, Bonferroni corrected. No significant difference was shown between T2DM-NC and HC with post hoc test, Bonferroni corrected. MMSE, Mini-Mental State Examination; MoCA, Montreal Cognitive Assessment; AVLT, Auditory Verbal Learning Test; ROCF, Rey-Osterrieth Complex Figure; WAIS, Wechsler Adult Intelligence Scale; DST, Digital Span Test; TMT, Trail Making Test; VFT, Verbal Fluency Test.

### Group Differences in WMV Among the Groups

The WMV of the whole brain of T2DM-MCI, T2DM-NC, and HC groups were analyzed. If statistical differences among the three groups were observed, then a *post hoc* test was performed. It was not surprising that obvious difference in WMV among these three groups were observed in 13 regions (*P* < 0.05, FDR corrected, Table 3). Subsequently, we performed a *post hoc* test and Bonferroni correction. Significant difference of regional WMV was observed in T2DM-MCI patients relative to T2DM-NC patients. Regions of the white matter atrophy were the left insula, posterior cingulate, and precuneus, right lateral orbitofrontal gyrus, pars orbitalis gyrus, rostral middle frontal gyrus, and temporal pole (Bonferroni-correction, *P* < 0.017) (Table 3). However, there was no significant difference between T2DM-NC and HC groups (Table 3). In addition, the included T2DM patients had no obvious white matter hyperintensity lesions on T2 FLAIR, which indicates that brain white matter atrophy rather than cerebrovascular lesions may substantially mediate the relationship between T2DM and cognitive impairment.

**TABLE 3:** Differences in the white matter volume among T2DM-MCI, T2DM-NC, and HC groups.

Regions	White matter volume			*F*-values	ANOVA
	HC	T2DM-NC	T2DM-MCI		(*P*-values)
Left isthmus cingulate	3,931.40 ± 569.78	3,819.84 ± 570.83	3,470.82 ± 603.25	5.122	0.048
Left lateral orbitofrontal gyrus	6,700.99 ± 838.21	6,702.80 ± 570.27	6,267.02 ± 770.28	3.500	0.042
Left posterior cingulate	4,379.01 ± 610.81	4,652.48 ± 500.46	4,231.84 ± 441.51	5.010	0.027*
Left precuneus	9,442.53 ± 1,743.27	9,833.90 ± 1,242.31	8,847.39 ± 1,264.13	3.593	0.048*
Left insula	8,918.20 ± 946.21	9,140.63 ± 746.48	8,618.25 ± 695.40	3.194	0.046*
Right lateral orbitofrontal gyrus	6,569.96 ± 721.97	6,734.11 ± 641.00	6,196.94 ± 765.54	4.492	0.049*
Right pars orbitalis	1,134.16 ± 232.18	1,209.77 ± 168.79	1,063.01 ± 187.21	4.128	0.044*
Right rostral middle frontal gyrus	12,509.42 ± 2,160.15	12,959.35 ± 1,604.56	11,768.02 ± 1,791.73	3.116	0.049*
Right temporal pole	687.63 ± 128.12	745.50 ± 113.06	669.06 ± 119.51	3.290	0.049*

Values are the mean ± standard deviation. The comparisons of white matter volume of each region among three groups were performed with ANOVA. The level of significance for intergroup differences was set at P < 0.05, false discovery rate (FDR) corrected. * Denotes P < 0.017 (0.05/3) T2DM-MCI vs. T2DM-NC with post hoc test, Bonferroni corrected. No significant difference was shown between the T2DM-NC group and HC group with post hoc test, Bonferroni corrected.

### WMV in Relation to Cognitive Performance in T2DM Patients

**FIGURE 1:**
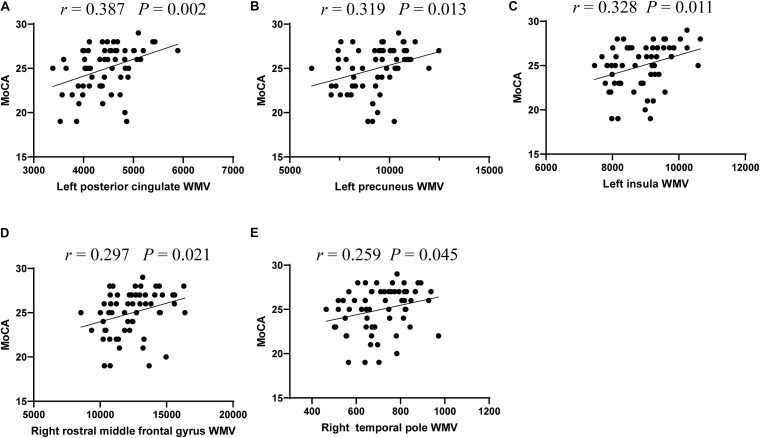
The significant correlations between white matter volume and cognitive performance in T2DM patients. Correlations between MoCA scores and white matter volume of five regions, including left posterior cingulate **(A)**, left precuneus **(B)**, and left insula **(C)**, right rostral middle frontal gyrus **(D)**, and right temporal pole **(E)**.

Based on the WMV and cognition scores with significant intergroup differences, we explored the relationship between WMV of these regions and the neuropsychological testing scores in all T2DM patients. The results revealed that MoCA scores were significantly correlated with the WMV of the left posterior cingulate (*r* = 0.387, *P* = 0.002), precuneus (*r* = 0.319, *P* = 0.013), insula (*r* = 0.328, *P* = 0.011), right rostral middle frontal gyrus (*r* = 0.297, *P* = 0.021), and temporal pole (*r* = 0.259, *P* = 0.045) (Figure 1).

### Discriminative Ability of White Matter Atrophy for MCI Detection in T2DM Patients

**FIGURE 2:**
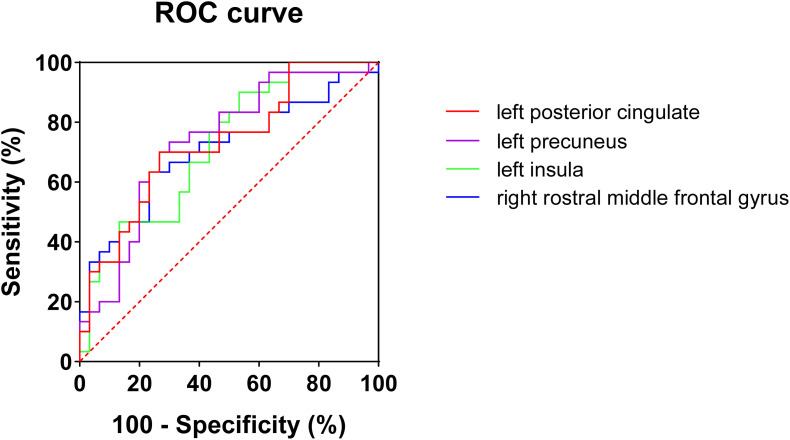
ROC curves of white matter volume of four single regions for mild cognitive impairments in T2DM patients. No significant difference (*Z* = 0.597, *P* = 0.551) was found between minimum AUC (right rostral middle frontal gyrus) and the maximum AUC (left precuneus).

Considering that the WMV of eight brain regions significantly correlated with MoCA scores, we evaluated the discriminative ability of the white matter atrophy of these regions for MCI detection in T2DM patients. The ROC results showed that, when used alone, the WMV of left posterior cingulate, precuneus, insula, and right rostral middle frontal gyrus had high diagnostic value for MCI detection in T2DM patients (AUC > 0.7, Table 4), among which the value of WMV of left precuneus was the highest (AUC: 0.736, sensitivity: 70%, specificity: 73.33%). While the other region, right temporal pole, demonstrated little significance to identify T2DM-MCI patients (AUC = 0.686, Table 4). Furthermore, there was no significant difference between the minimum AUC (right rostral middle frontal gyrus) and the maximum AUC (left precuneus) among these four single markers (*Z* = 0.597, *P* = 0.551), which had high diagnostic value for MCI detection in T2DM patients (Figure 2). Above all, each WMV of these four regions, including left posterior cingulate, precuneus, insula, and right rostral middle frontal gyrus, could be the independent imaging biomarkers for MCI identification in T2DM patients.

**TABLE 4:** Diagnostic values of single imaging marker for MCI in T2DM patients.

Regions (white matter volume)	SEN (%)	SPE (%)	+LR	−LR	Yden	Cutoff	AUC
Left posterior cingulate	70.00	73.33	2.62	0.41	0.4333	4,419.8	0.729
Left precuneus	70.00	73.33	2.62	0.41	0.4333	9,413.7	0.736
Left insula	90.00	46.67	1.69	0.21	0.3667	9,283	0.712
Right rostral middle frontal gyrus	63.33	76.67	2.71	0.48	0.4000	12,077.7	0.704
Right temporal pole	70.00	66.67	2.10	0.45	0.3667	702.7	0.686

**SEN, sensitivity; SPE, specificity; +LR, positive likelihood ratio; −LR, negative likelihood ratio; Yden, Youden index; AUC, areas under the curves.**

## Discussion

In this study, cognitive function assessed by various neuropsychological tests showed that T2DM-MCI patients performed worst among these three groups, while there was no significant difference between T2DM-NC patients and HC. Subsequently, T2DM-MCI patients had white matter atrophy of several regions, and T2DM-NC patients did not show any white matter deficits. In particular, WMV of five regions including the left posterior cingulate, precuneus, insula, right rostral middle frontal gyrus, and temporal pole were significantly correlated with MoCA scores in T2DM patients. More importantly, we identified that each WMV of left posterior cingulate, precuneus, insula, and right rostral middle frontal gyrus could be the independent imaging biomarkers for T2DM-associated MCI detection.

To explore the white matter atrophy due to T2DM and T2DM-associated cognitive impairments, we compared the difference of WMV among T2DM-MCI, T2DM-NC, and HC groups. As expected, the WMV was considerably different in some regions among these three groups with FDR correction. After a *post hoc* test and Bonferroni correction, T2DM-MCI patients had white matter atrophy in the left insula, posterior cingulate, and precuneus, right lateral orbitofrontal gyrus, pars orbitalis gyrus, rostral middle frontal gyrus, and temporal pole, when compared with T2DM-NC patients without clinically confirmed cognitive impairment. Additionally, the T2DM-NC patients did not show significant atrophy in brain white matter compared with the HC. These results indicated that the observed atrophy in brain white matter is strongly associated with cognitive impairment in T2DM patients. Previous studies reported that T2DM was associated with white matter loss mainly in frontal and temporal white matter, which were partly in line with our findings (Chen et al., 2012; Moran et al., 2013). However, some other studies revealed that individuals with T2DM had reductions in hippocampus volumes but superior temporal gyrus had no volume loss, which were not in accordance with our results (Korf et al., 2006; Gold et al., 2007; Cherbuin et al., 2012). In addition, we found that some limbic structures and precuneus, which have been reported associated with cognitive functions (Cavanna and Trimble, 2006; McCrimmon et al., 2012; Hoogenboom et al., 2014), had obviously reduced WMV. The reason of the inconsistent results may be associated with the cognitive state of the included T2DM patients. T2DM patients included in these previous studies were in different stages of diabetes-associated cognitive impairment, whereas in our study, we enrolled T2DM-MCI, the early stages of cognitive impairment in T2DM patients, and T2DM-NC without clinically confirmed cognitive impairments to provide early signs of cognitive dysfunction, which may help clinicians with the earlier prevention of severe cognitive decline in T2DM patients.

The MoCA scores, which served as an index of the cognitive status of the patients, are known to distinguish patients with MCI form the normal population (Petersen et al., 1999). MoCA has shown higher sensitivity in detecting cognitive decline than the MMSE (Nasreddine et al., 2005). Our results revealed that no significant differences were shown in MMSE scores among these three groups, while MoCA scores were significantly higher in HC when compared with T2DM-MCI and T2DM-NC patients, and T2DM-NC patients performed better than T2DM-MCI patients. Furthermore, WMV of five brain regions significantly correlated with the MoCA scores, mainly located in frontotemporal lobe and limbic system, which have been implicated in memory and learning (McCrimmon et al., 2012). Among them, the correlation coefficient of the left posterior cingulate WMV was higher than others. Cingulate gyrus, as one of the important limbic structures, has been reported to be disrupted in T2DM patients (Yau et al., 2010; Hoogenboom et al., 2014; Tan et al., 2016; Nouwen et al., 2017; Groeneveld et al., 2018; Yu et al., 2019). More importantly, its disruption was associated with impairments in cognitive functions including memory, psychomotor speed performance, and executive function in T2DM patients (Hoogenboom et al., 2014). Previous studies have discovered reduced functional connectivity in the default mode network between posterior cingulate gyrus and left medial frontal gyrus in middle-aged T2DM patients. Moreover, reduced FA in the cingulum was correlated with lower functional connectivity between cingulate and medial frontal gyrus (Hoogenboom et al., 2014). Moreover, the white matter loss was also reported in cingulate gyrus attributed to T2DM (Chen et al., 2012). More interestingly, our study also showed that the WMV of left posterior cingulate had a high discriminative ability for T2DM patients with MCI. Thus, the white matter disruption of cingulate gyrus has a possible relationship with cognitive impairments at the early stage in T2DM patients.

In addition, to find non-invasive biomarkers for early detection of cognitive impairment in T2DM patients, we evaluated the discriminative ability of these five regions for MCI in T2DM patients. Four regions had high diagnostic value for MCI detection in T2DM patients by performing ROC analysis of a single region, among which the value of left precuneus was the highest. The precuneus has been reported to play a central role in a wide spectrum of highly integrated tasks, including visuo-spatial imagery (Simon et al., 2002; Malouin et al., 2003; Wenderoth et al., 2005), episodic memory retrieval (Platel et al., 2003; Addis et al., 2004; Lundstrom et al., 2005), and self-processing operations (Vogeley et al., 2004; den Ouden et al., 2005; Cavanna and Trimble, 2006). Moreover, the white matter disruption and reduced functional connectivity of the precuneus were found in T2DM patients and significantly correlated with disease duration (Zhou et al., 2010; Hsu et al., 2012; Wood et al., 2016). Thus, the white matter atrophy of precuneus could be a part of mild cognitive dysfunction in T2DM patients. Then, we compared the AUC of these four regions’ WMV, which presented high diagnostic value for MCI detection in T2DM patients. The results showed that no significant difference was found in maximum AUC relative to the minimum AUC among the four regions’ WMV. Therefore, each WMV of the four regions, including left posterior cingulate, precuneus, insula, and right rostral middle frontal gyrus, could be the independent imaging biomarker for detecting mild cognitive dysfunction in T2DM patients.

There are also several limitations in our study. First, this current research is a cross-sectional study with a relatively small sample size, and a longitudinal study with a larger sample size is needed in the future to test whether these WMV atrophy could predict the development of cognitive impairment in T2DM patients. Second, although we enrolled age-, sex-, and education-matched HC, T2DM-MCI patients had higher BMI index than HC. However, there were no significant difference in BMI between T2DM-MCI and T2DM-NC patients, with the result that the effect of BMI on the results should be little. Third, although education has been used as a control variable in this study, the participants with different levels of educational achievement may show different effects. Fourth, we only focused on the white matter atrophy in T2DM patients with MCI. Some microstructural damage of white matter could not be detected on conventional MR images. Exploring the WMV, integrity, and network connection in the same participants will provide more details on the pathophysiological mechanism of white matter. Thus, multimodal imaging methods are needed to investigate the white matter damage in T2DM association with cognitive deficits in future.

## Conclusion

In conclusion, the present study revealed that T2DM-NC patients did not show any white matter damage, and T2DM-MCI patients showed significantly reduced WMV in several regions, which suggests that significant white matter atrophy occurred in MCI stage in T2DM patients. In particular, MoCA scores in T2DM patients had significant correlations with the white matter loss of the five regions mainly in the frontotemporal lobe and limbic system. Moreover, each WMV of the four regions, including left posterior cingulate, precuneus, insula, and right rostral middle frontal gyrus, could be the independent imaging biomarker for T2DM-associated cognitive impairment at the early stage and play an important role in its pathophysiological mechanism.

## Data Availability Statement

The original contributions presented in the study are included in the article/supplementary material, further inquiries can be directed to the corresponding author/s.

## Ethics Statement

The studies involving human participants were reviewed and approved by the Research Ethics Committee of Southwest Hospital, Army Medical University, Chonqing, China. The patients/participants provided their written informed consent to participate in this study.

## Author Contributions

XC, JW, and KX conceived and designed the study. CL and YL collected the data of MRI, clinical, and neuropsychological test. ZZ and RJ analyzed MRI data. KL and HT performed statistics. YG and JZ performed figure and table preparation. XC and KL wrote the manuscript. JS and QZ edited the manuscript. All authors revised the manuscript, and read and approved the submitted version.

## Conflict of Interest

The authors declare that the research was conducted in the absence of any commercial or financial relationships that could be construed as a potential conflict of interest. The reviewer XY declared a past co-authorship with the authors CL, ZZ, and JW to the handling editor.
